# Patient Attitudes About Viewing Their Radiology Images Online: Preintervention Survey

**DOI:** 10.2196/12595

**Published:** 2019-07-18

**Authors:** Ciarra Halaska, Peter Sachs, Kate Sanfilippo, Chen-Tan Lin

**Affiliations:** 1 UCHealth Aurora, CO United States; 2 Department of Radiology University of Colorado School of Medicine Aurora, CO United States; 3 University of Colorado School of Medicine Aurora, CO United States

**Keywords:** connected health, electronic health records, information transparency with patients, online patient-physician communication, online patient portal, radiology images, second opinion, social media, test result management

## Abstract

**Background:**

Although patient data is available through electronic portals, little information exists about the benefits and/or challenges of providing patients with online access to their radiology images.

**Objective:**

The aims of this quality improvement project were to understand patient attitudes toward being able to view their radiology images online and determine how information should be presented to ensure the images are helpful to the patients, rather than causing confusion and anxiety.

**Methods:**

An online survey of consumers was conducted to evaluate attitudes toward online access to personal radiological images.

**Results:**

A total of 105 responses were received from 686 community members (15.3%). Of 105 consumers, 94 (89.5%) reported a desire to have access to the radiology images within their online patient portal; 86.7% (91/105) believed it would help them better understand their medical conditions and 81.0% (85/105) said this would help them feel more in control of their care. Most respondents (74/105, 70.5%) said it would help them feel reassured that their doctor was doing the right thing, and 63.8% (67/105) said it would increase their level of trust in their doctor. Among surveyed patients, 78.1% (82/105) valued viewing their radiology images online, while 92.4% (97/105) valued their online radiology reports. Most patients (69/105, 65.7%) wished to discuss their results with their ordering clinician, 29.5% (31/105) wished to discuss with their interpreting radiologist, and 3.8% (4/105) wished to share their images on social media. The biggest potential concern among 23.8% (25/105) was that the images would be confusing.

**Conclusions:**

A large majority of surveyed patients desired the ability to view their radiology images online and anticipated many benefits and few risks. Health care organizations with electronic health records and online patient portals should consider augmenting their existing portals with this highly desired feature. To avoid the biggest patient concern, radiology reports should accompany images. Patients wanted to discuss their results with their ordering physician and their interpreting radiologist. Some even would like to share results on social media. Further research on the actual experience with such a tool will be needed.

## Introduction

Online patient portals linked to electronic health records (EHRs) typically offer tools such as online communication between patient and clinician and patient access to portions of their medical record data, which includes test results, radiology reports, and pathology reports. Patients have long expressed a desire to view their medical reports, with expected benefits and few perceived risks [1]. More recently this includes viewing reports via online tools [2-6]. The nationwide Open Notes initiative took this further in 2011 [7,8]. Surveys demonstrated patient interest in viewing their radiology reports and images, and patients perceive there are potential benefits from doing so [9,10]. Although other institutions, such as the Mayo Clinic and the Department of Veterans Affairs have image-enabled their patient portals, to our knowledge there have been few published surveys regarding patient perceptions of the advantages of being able to view their own radiology images online. Greco et al [11] conducted a limited survey of patients viewing images in a personal health record (PHR) and found they responded favorably to having access to their images while expressing a high level of concern for the privacy of their health information.

A group composed of clinical and imaging informatics specialists focused on improving health care technology developed a method to offer patients the ability to view radiology images associated with their radiology report in the online patient portal. However, we were uncertain about how this new tool might be received by the patient population. Therefore, we decided to solicit opinions from an online community in order to gauge interest prior to implementation.

## Methods

### Survey Design

We designed a survey to evaluate attitudes of patients regarding the ability to view their own radiological images online; it was intended to serve as a baseline survey to be refielded postimplementation (see Multimedia Appendix 1 for the survey instrument). The project was initiated by an information technology program manager (KS), senior market research analyst (CH), physician imaging informaticist (PS), and chief medical information officer (C-TL). We conducted this project as market research and quality improvement with an established panel of volunteer community members across Colorado; therefore, the study was exempt from review by the institutional review board.

### Target Population

This was a preintervention survey of 686 community members, many of whom were patients at UCHealth. The UCHealth Insights Community exists to collect community feedback in order to help UCHealth elevate how health care providers interact with the community and continuously evolve the health care experience and reflects the demographics of the health care decision makers in Colorado. Members are recruited via community partnerships, social media, and the UCHealth website, including My Health Connection (UCHealth’s online patient portal). The screening criteria required for community members to join the Insights Community are that they must live in Colorado, cannot work in health care, must be involved in their household’s health care decisions, must have health insurance, and cannot participate in more than three online research communities. There were no additional participation criteria set for this quality improvement project. This online feedback community is hosted by a commercial entity (My-Take.com).

### Recruitment

Potential participants received one email invitation describing the purpose of the survey and the opportunity to participate and one email reminder if they had not yet completed the survey. With previous surveys in this community, further reminders did not increase response rates. The survey was available for six days. Participants were reminded that their participation would earn them an entry into a monthly prize drawing, in accordance with the UCHealth Insights Community site policy. This prize drawing was standard procedure for the Insights Community independent of this particular survey. Participants from the Insights Community were surveyed in July 2018.

### Statistical Analysis

Descriptive information regarding demographics of participants was collected along with behavioral components related to this survey—specifically whether they were a UCHealth patient, had used radiology services in the past year, and had used an online patient portal at that time to manage their health. To gauge interest in the option to have radiology images provided online within their patient portal, we examined the distribution of responses to questions across a range of categorical and ordinal variables within the survey. After reading through all responses, open-ended responses were coded manually, sorting each response into a bucket. Buckets were developed by determining which themes came up most frequently. One response could be coded into multiple buckets, for example “Understanding the results and why my doctor is making recommendations. Sharing with surgeons, etc” was coded as both “increase understanding” and “conveniently see/share results.” We rated responses to quantitative questions about value from 1=not at all valuable to 5=extremely valuable. We rated responses to questions about level of agreement from 1=strongly disagree to 5=strongly agree. In order to report the survey results as percentages, Likert scores are reflected as top 2 box scores or scores of a 4 or 5 on these 5-point scales. We conducted all statistical analyses using Q Research Software version 5.4.5.0 (Displayr).

## Results

### Target Population

We received a total of 105 responses from a target population of 686 members who were invited to participate in this research, yielding a 15.3% response rate. The mean age of the sample was 50.37 (SD 15.08) years, and most respondents were female (74/105, 70.5%). A majority of the sample identified as white (85/105, 81.0). All respondents lived in Colorado, and 59.0 (62/105) of respondents were UCHealth patients. Over half (60/105, 57.1%) had used radiology services in the past year, and approximately half of those respondents (31/60, 52%) had viewed their radiology report online. Most participants (80/105, 76.2%) used their online patient portal regardless of where they received care. The sample size was too small to detect differences in responses from those with and without portal accounts. There were no significant demographic differences between responders and nonresponders.

### Survey Results

When asked whether they would want access to future radiology images online, 89.5% (94/105) said yes. Of these 94 respondents, 33 (35%) explained in the open-ended follow-up that this would help them to understand their radiology results at a higher level, especially when paired with their radiology report, and 27 respondents (29%) reported they desired the convenience of having access to their images online; 89.5% (94/105) of responders liked the idea of viewing their images in the online patient portal. The 10.5% (11/105) who disliked the idea were asked why. Open-ended responses indicated that people did not see a need for this offering (3/11) and would rather view and discuss their images in person with their doctor (4/11).

When assessing the value of online access to radiology images compared to radiology reports, 78.1% (82/105) found value in having online access to radiology images compared with 92.4% (97/105) who found value in radiology reports.

Participants rated their level of agreement with various statements regarding online access to radiology images. The full distribution can be found in Table 1. Access to online radiology images received high top 2 box scores for improved patient-doctor relationships: increased levels of trust (67/105, 63.8%), feeling reassured that their doctor is doing the right thing (74/105, 70.5%), and being able to better follow their doctor’s recommendations (74/105, 70.5%). Participants also agreed that viewing their images would help them to understand their medical conditions (91/105, 86.7%) and feel more in control of their health care (85/105, 81.0%). Few participants reported they were concerned with finding errors (10/105, 9.5%) or being worried (10/105, 9.5%) or confused (9/105, 8.6%) when viewing their images.

Anticipating that patients would have questions about their images, we asked participants how they would use their online images, and with whom they would most like to discuss their images; responses are shown in Table 2. Of the respondents, 80.0% (84/105) reported they would like the ability to share their images with their doctor, 78.1% (82/105) desired to save a copy directly from their online portal, 62.9% (66/105) would share their images with other doctors for a second opinion, and 3.8% (4/105) of our respondents indicated they would be interested in sharing their radiology images on social media.

Results to the voluntary open-ended question regarding the benefits of viewing images online are shown in Table 3. The convenience of seeing, saving, and sharing their images was cited by 48.6% of respondents (51/105); 46.7% (49/105) indicated that it would increase their understanding.

It would help me understand my condition better, and it would give me more time to formulate questions for my doctor.

**Table 1:** Distribution of agreement with statements regarding online viewing of images (N=105).

Statement: I believe that viewing my radiology images online would cause me to...	Agreement with statement, scored on a 5-point scale, n (%)		
	Disagree (bottom 2)	Neutral (middle 3)	Agree (top 2)
...feel confused or have a lot of questions.	70 (66.7)	26 (24.8)	9 (8.6)
...worry more.	74 (70.5)	21 (20.0)	10 (9.5)
...find errors in my radiology reports.	68 (64.8)	27 (25.7)	10 (9.5)
...trust my doctors more.	15 (14.3)	23 (21.9)	67 (63.8)
...feel reassured.	8 (7.6)	23 (21.9)	74 (70.5)
...better follow recommendations.	13 (12.4)	18 (17.1)	74 (70.5)
...feel more in control.	6 (5.7)	14 (13.3)	85 (81.0)
...better understand my medical condition.	4 (3.8)	10 (9.5)	91 (86.7)

**Table 2:** Participant responses to the question: What would you do with your online images? (N=105)

Response	Agreement with statement, n (%)
Share them with my primary care doctor, if they don’t have them already	84 (80.0)
Save a copy for my records	82 (78.1)
Share them with other doctors for a potential second opinion	66 (62.9)
Share them on social media	4 (3.8)
Other	8 (7.6)
None of the above	6 (5.7)

**Table 3:** Benefits described by participants when asked the open-ended question: What would the benefits be of viewing your radiology images online? (N=105)

Benefit of viewing images	Value n (%)
Conveniently see, save, and share images	51 (48.6)
Increase understanding	49 (46.7)
Increase level of control	12 (11.4)
Use for second opinion from another doctor	10 (9.5)
Formulate/ask questions	9 (8.6)
Cool/curiosity	7 (6.7)
Avoid follow-up appointment	3 (2.9)

**Table 4:** Participant concerns expressed in response to the open-ended statement: Please explain any concerns about viewing your radiology images online. (N=105)

Concern	Value n (%)
No concerns	50 (47.6)
Would cause confusion	25 (23.8)
Security/privacy concerns	19 (18.1)
Can’t ask questions or get answers quickly	19 (18.1)
Mistakes (wrong images could be uploaded)	3 (2.9)

Participants also expressed how tedious it currently is to obtain a copy of their radiology images.

It saves me the hassle of filling out a form, sending an email, making a trip to the doctor’s office, making four phone calls, and spending an hour on hold to make sure my specialist has everything they need for my appointment. That is what I had to do for the images, and the report still didn't come across, so when I had a few moments I logged into the portal and printed out the report to bring with me. I would much rather take the self-service route to save me the time and stress of getting images from the facility to the doctor, plus I would have the opportunity to see the images myself and better understand the issue when discussing it with my doctor.

Participants concerns when asked the voluntary open-ended question about viewing their images online are shown in Table 4. Of the respondents, 47.6% (50/105) said they had no concerns. Concerns regarding confusion (23.8%, 25/105) and security/privacy (18.1%, 19/105) were the most prevalent.

Not understanding what I'm looking at is a big [concern]. There is a reason that doctors go to school to learn how to read images. This is why the radiology report is also essential. It ties the two together.

I worry a little about security but would hope that you guys provide encrypted portals, passwords, etc.

In response to question 11, 65.7% of respondents (69/105) reported they would prefer to discuss their images with the doctor who referred them to radiology, and 29.5% (31/105) would rather discuss with the radiologist who wrote their report.

The final question of the survey asked participants to rate the idea of being able to view their radiology images in their online patient portal. The response from 86.7% (91/105) was favorable, which resulted in a top 2 box score.

## Discussion

### Principal Findings

Our main finding is that our respondents had a high level of interest in viewing their images with a surprisingly low level of concern about possible confusion. Overall, patients preferred to view their images alongside the reports, as viewing images without the report was seen as potentially confusing. Patients also had a strong interest in being able to download their images from the portal. Patients expressed frustration with the current process that requires they come to the radiology file room, request a CD of images, then physically transport the CD to the recipient, who may not have a CD drive. A small percentage of patients also expressed an interest in sharing their images on social media.

Multiple previous reports, including the Open Notes literature [7,8], have described the benefits of patient access to their medical record via an EHR-based patient portal in both the ambulatory and inpatient settings. These include a sense of increased feelings of empowerment and autonomy, “including control, understanding, reassurance, and following recommendations” [12,13]. Patients have also described feeling an increased ability to coordinate their own care and remember important health care tasks and an improved sense of participating in their own care [4]. Anticipated risks such as increased patient worry and increased provider workload have not been borne out [12]. Interestingly, such access has not been shown to improve health status [3].

Radiology has also embraced the value to both patients and the specialty of increased visibility of imaging reports to and greater interaction with patients, with the Radiology 3.0 initiative serving as a foundation [14]. This has focused on direct contact with patients via consultation clinics or widespread face-to-face transmission of reports to patients by interpreting radiologists [15]. To date, this has gained the most traction in the breast imaging sphere [16]. Patients have indicated their desire to view their reports quickly and in understandable detail [5]. Multiple reports have demonstrated that patients value online access to their imaging reports and that doing so does not increase workloads for the ordering providers [17]. Interestingly, patients have indicated that although they value direct access to their reports, they prefer to first hear the results from their ordering provider and prefer to review their images with their provider when receiving the results [9,18]. While some patients do not wish to view their images, for those who do the experience can offer a powerful way to connect with their illness, with their provider, and should be offered [10].

### Limitations

This baseline survey had a low response rate (15%) among community members, consisted mostly of female respondents (70%), and was performed at one institution in one geographical location, which may limit the generalizability of these findings. However, the response rate was typical of response rates in other market research surveys. For example, Greco et al [11] had a 19.6% response rate in their survey of patients using a PHR for image exchange and viewing. In question 6, we asked about the value of viewing reports and viewing images and did not give an option for viewing images with reports. This may have been confusing to respondents and makes interpretation of the responses potentially ambiguous. Our community members expressed a high level of interest in radiology images, which may reflect members who were patient portal users (76%). However, the level of high interest is similar to the greater than 80% interest in images seen in previous reports [9].

### Comparison With Prior Work

A consortium of four academic institutions (Mt. Sinai New York, University of California San Francisco, Mayo Clinic, and University of Maryland) collaborated to provide their patients with a personal heath record (PHR) that included image viewing using the Radiological Society of North America’s Image Share Network [11]. We note that, unlike our integrated project, this publicly available image viewing site is separate from their health care organization’s patient portal. Their survey of users focused primarily on satisfaction with the specific technologies available (eg, CD or internet) for image downloading and viewing. They found that 96.5% responded favorably to having access to their images and imaging reports and 78% viewed their images independent of their providers. Patients were concerned about the privacy of the PHR (67% to 78%), but other potential concerns were not explored. They also did not assess patient preference for viewing their images with others (provider, family, social media) nor did they solicit additional feedback as we did.

### Future Directions

Our health system is in the process of implementing radiology image viewing through the online patient portal and plans to report on the findings and experiences of patients actually viewing their images online. We will enable image viewing using the currently available enterprise viewer provided with our Picture and Archiving Communication System. This is a view-only system currently optimized for desktop viewing. Based on the survey feedback, patients will be able to download and/or share their images using an existing commercial image exchange tool, which is currently used by the health system for image exchange between health care institutions and providers but not yet embedded in the patient portal. This functionality will allow patients to upload and download their images via CD, thumb drive, and the cloud. The enterprise is also currently evaluating mobile friendly viewers that will further enhance and simplify the patient experience. An important means for supporting patient’s questions about radiology images will be a tool allowing easy communication between the patient and the ordering provider as well as the interpreting radiologist (personal communication by Alexander J. Towbin, MD, on 10/28/2018). As momentum grows for immediate access by patients to all their images and reports, the ability to communicate directly with the interpreting radiologist may alleviate some ordering providers concern about potential patient anxiety and possible increased burden on them to respond to questions [19,20].

The interest in sharing radiology images on social media is worthy of exploration in future work. Possible motivations include ultimate transparency (eg, “I want everyone to know what I have”), seeking a second opinion (eg, “Maybe if I tweet this, some radiologists somewhere will have a different idea”), finding a community (eg, “Maybe someone else out there has the same thing and we can be friends”), or perhaps a cool factor (ie, “No one else can do this, look at me!”).

### Conclusions

A large majority of surveyed patients desired the ability to view their radiology images online and anticipated many benefits and few risks. Health care organizations with EHRs and online patient portals should consider augmenting their existing portals with this highly desired feature. Further research on actual experience with such a tool will be needed and helpful.

## Appendix

Multimedia Appendix 1 Preintervention survey.

## Abbreviations

EHR: electronic health record
PHR: personal health record
