# Supplementary material for: Patient Attitudes About Viewing Their Radiology Images Online: Preintervention Survey
Source: J Med Internet Res. 2019 Jul 18;21(7):e12595. doi: 10.2196/12595 (PMC6670277; doi:10.2196/12595)
Supplement: Multimedia Appendix 1 [file jmir_v21i7e12595_app1.pdf]

**APPENDIX 1:** Preintervention Survey

**Survey Design:**

*Thank you for taking the time to provide your feedback. This survey will take about five minutes of your time.*

Please respond to the following statements:

Radiology reports describe the findings in the radiology images.

Radiology image(s) include X-rays, CT scans, MRI scans, and ultrasounds.

1. Are you a current UCHealth patient?
   a. Yes
   b. No

2. (If yes to #1) Do you utilize MyHealthConnection? This is the online patient portal where you are able to view your test results, refill prescriptions, etc.
   a. Yes
   b. No
   (If no to #1) Do you utilize an online patient portal with your current health care provider where you are able to view your test results, refill prescriptions, etc.?
   c. Yes
   d. No

3. In the past year, have you had any radiology images taken for any reason? This includes X-rays, CT scans, MRI scans, and ultrasounds.
   a. Yes
   b. No

4. (If yes to #3) Have you ever viewed your radiology report in My Health Connection or any other online patient portal?
   A radiology report describes the findings in the radiology images.
   a. Yes
   b. No

5. (To all respondents) If you were to have radiology images taken...
   In addition to viewing your radiology report, would you also like to be able to view your radiology image(s) within your online patient portal?
   Radiology reports describe the findings in the radiology images.
   Radiology image(s) include X-rays, CT scans, MRI scans, and ultrasounds.
   a. Yes (please explain why)
   b. No (please explain why)

6. Please rate the value of viewing each of the following within your online patient portal (1=not at all valuable, 5=extremely valuable):
   Radiology reports describe the findings in the radiology images.
   Radiology image(s) include X-rays, CT scans, MRI scans, and ultrasounds.
   a. Radiology report
   b. Radiology images

7. Please rate the following statements (1=Strongly Disagree, 5=Strongly Agree):
   "I believe that viewing my radiology images online would cause me to…
   (randomize order)
   a. …worry more."
   b. …feel confused/have a lot of questions."
   c. …feel more in control
   d. …better understand my medical condition."
   e. …feel reassured."
   f. …better follow recommendations."
   g. …trust my doctors more."
   h. …find errors in my radiology reports."

8. Which of the following would you like to do with your radiology images if you had access to them online? Select all that apply.
   a. Share them with my primary care doctor if they don't have them already
   b. Share them with other doctors for a potential second opinion
   c. Share them on social media
   d. Save a copy for my records
   e. Other (please specify)
   f. None of the above

9. What would the benefits be of viewing your radiology images online? (open ended)

10. Please explain any concerns about viewing your radiology images online. (open ended)

11. If you had questions about your radiology images during/after viewing them online, who would you most want to discuss them with?
    a. The doctor who referred me to radiology
    b. The radiologist who wrote my radiology report
    c. Other (please specify)

12. Overall, please rate the idea of being able to view your radiology images in your online patient portal (1 = strongly dislike, 5 = like it a lot).

*Thanks again for your time in completing this survey!*
